# Association between Scale-Free Brain Dynamics and Behavioral Performance: Functional MRI Study in Resting State and Face Processing Task

**DOI:** 10.1155/2017/2824615

**Published:** 2017-12-21

**Authors:** Masato Kasagi, Zirui Huang, Kosuke Narita, Hitoshi Shitara, Tomokazu Motegi, Yusuke Suzuki, Kazuyuki Fujihara, Sean Tanabe, Hirotaka Kosaka, Koichi Ujita, Masato Fukuda, Georg Northoff

**Affiliations:** ^1^Department of Psychiatry and Neuroscience, Gunma University Graduate School of Medicine, 3-39-22 Showa-machi, Maebashi, Gunma 371-8511, Japan; ^2^Institute of Mental Health Research, University of Ottawa, Ottawa, ON, Canada K1Z 7K4; ^3^Department of Orthopaedic Surgery, Gunma University Graduate School of Medicine, Maebashi, Gunma 371-8511, Japan; ^4^Research Center for Child Mental Development, University of Fukui, Eiheiji-cho, Fukui 910-1193, Japan; ^5^Department of Diagnostic Radiology and Nuclear Medicine, Gunma University Graduate School of Medicine, Maebashi, Gunma 371-8511, Japan; ^6^Center for Cognition and Brain Disorders, Hangzhou Normal University, Hangzhou 311121, China; ^7^Zhejiang Key Laboratory for Research in Assessment of Cognitive Impairments, Hangzhou Normal University, Hangzhou 310015, China; ^8^Taipei Medical University, Graduate Institute of Humanities in Medicine, Taipei, Taiwan; ^9^Brain and Consciousness Research Center, Taipei Medical University-Shuang Ho Hospital, New Taipei City, Taiwan

## Abstract

The scale-free dynamics of human brain activity, characterized by an elaborate temporal structure with scale-free properties, can be quantified using the power-law exponent (PLE) as an index. Power laws are well documented in nature in general, particularly in the brain. Some previous fMRI studies have demonstrated a lower PLE during cognitive-task-evoked activity than during resting state activity. However, PLE modulation during cognitive-task-evoked activity and its relationship with an associated behavior remain unclear. In this functional fMRI study in the resting state and face processing + control task, we investigated PLE during both the resting state and task-evoked activities, as well as its relationship with behavior measured using mean reaction time (mRT) during the task. We found that (1) face discrimination-induced BOLD signal changes in the medial prefrontal cortex (mPFC), posterior cingulate cortex (PCC), amygdala, and fusiform face area; (2) PLE significantly decreased during task-evoked activity specifically in mPFC compared with resting state activity; (3) most importantly, in mPFC, mRT significantly negatively correlated with both resting state PLE and the resting-task PLE difference. These results may lead to a better understanding of the associations between task performance parameters (e.g., mRT) and the scale-free dynamics of spontaneous and task-evoked brain activities.

## 1. Introduction

Studies using functional magnetic resonance imaging (fMRI), magnetoencephalography (MEG), and electroencephalography (EEG) have shown that spontaneous brain activity can be characterized by scale-free dynamics. Scale-free dynamics can be revealed by scaling analysis, with which the fluctuations of a parameter as a function of the scale at which the parameter is evaluated can be quantified. Furthermore, the scale-free dynamics of brain activity can be indexed using the power-law exponent (PLE), that is, the power spectrum with the formula power ∝ 1/frequency *β*, where *β* is the PLE [1–8]. The properties of scale-free dynamics are shared by many systems found in nature [4, 9] and, most importantly, have also been observed in neural activity across many different spatiotemporal scales: from neurotransmitter release [10], neuronal spike trains [11], network firing rates [12], field potentials [4, 6, 7, 13, 14] to fMRI signals [2, 4, 15, 16]. Most recently, the properties of scale-free dynamics have been found in various human behaviors [3, 17] including motor behavior [7, 18, 19], perception [20], music composition [21], consciousness [22–24], development and maturation [25, 26], personality dimensions such as trait impulsivity [27] and extraversion [28], and self-consciousness [16]. These findings suggest a close relationship between scale-free dynamics during spontaneous brain activity and those during behavioral performance. However, studies explicitly focusing on the relationship between scale-free dynamics during spontaneous brain activity (specifically in the infraslow frequency range as measured by fMRI) and behavioral measures during a task are still lacking.

Studies on different psychiatric disorders such as anxiety [29], autism [30], depression [31], and schizophrenia [32] revealed changes in scale-free dynamics during spontaneous or task-state brain activity. These changes indicate an association between scale-free dynamics and face processing, which remains to be tested on a healthy brain. It is likely that scale-free dynamics during spontaneous and task states are not only affected by emotions but are also associated with behavioral performance parameters (e.g., reaction time) [3, 17].

The aim of our study was to investigate firstly the modulation of spontaneous activity PLE using a face processing task and secondly the association of PLE during both spontaneous and task-evoked activities with behavioral measures, for example, mean reaction time (mRT). To this end, we conducted an fMRI investigation of both spontaneous activity (resting state) and task-evoked activity (task state) using a face processing paradigm in a block design described in our previous report [32]. More specifically, we aimed for the following: (i) determination of brain regions showing an increase or a decrease in blood-oxygen-level-dependent (BOLD) signals between face processing and control conditions, (ii) determination of PLE in both the resting state and task state in the brain regions associated with face processing, and (iii) correlation of PLE in both the resting and task states with mRT in response to the recognition of an emotional face and control. On the basis of previous studies that showed a positive association between PLE in the cingulate cortex in the resting state and self-consciousness scale score [16] or depression rating scale score [31], we hypothesized that PLE during resting-state and task-state fMRI predicts the associated behavioral performance parameters (e.g., mRT), that is, a higher PLE in the resting state in the brain areas that contribute to responses during a task might be associated with the higher efficiency in processing of the task, which results in a shorter RT during the task.

## 2. Materials and Methods

### 2.1. Subjects

Fifteen participants (age range, 21–37 years; mean age, 26.5 ± 4.8 years; all males) were enrolled on the basis of the following exclusion criteria: history of neurological/psychiatric illness or a traumatic event (e.g., serious accident or physical/sexual abuse), chronic medication, chronic alcoholism, substance abuse, parental divorce, and history of psychiatric illness in first-degree relatives (see Table 1). For the evaluation of the past and current psychological conditions, the Structured Clinical Interview for DSM-IV Axis I Disorders [33] was used. All the subjects enrolled in this study were right-handed, as assessed using the Edinburgh Handedness Inventory [34]. All the subjects provided their written informed consent in accordance with the Declaration of Helsinki. The study protocol was approved by the Ethics Committee of Gunma University.

### 2.2. T1-Weighted Anatomical Imaging and Functional MRI Data Acquisition

Brain MRI was performed using Siemens 3-T Trio with a 12-channel head coil (Siemens, Erlangen, Germany) in Gunma University Hospital. High-resolution T1-weighted anatomical images [magnetization-prepared rapid acquisition with gradient echo (MP-RAGE) sequence] were acquired as follows: repetition time = 2000 ms; echo time = 2 ms; inversion time = 990 ms; flip angle = 90°; field of view (FOV) = 256 × 256 mm^2^; matrix size = 256 × 256; voxel size = 1 × 1 × 1 mm^3^. Functional images acquired in the resting and task states were collected using an echoplanar imaging (EPI) sequence (TR = 2500 ms, TE = 25 ms, flip angle = 90°, FOV = 220 × 220 mm^2^, matrix size = 64 × 64 with pixel dimensions of 3 × 3 mm^2^). Thirty-five axial slices with a thickness of 4 mm and an interslice gap of zero were acquired.

### 2.3. Experimental Design

**Figure 1:**
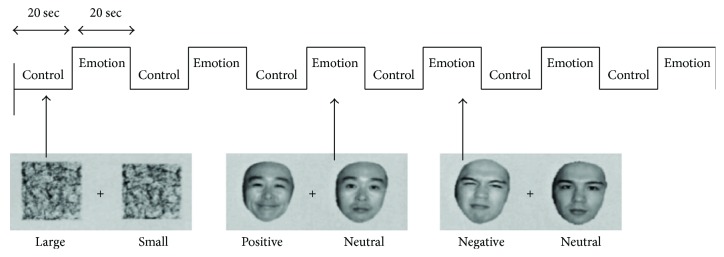
Paradigm of face processing task.

During the experiment, fMRI recordings were taken in both the resting state for 4 min and the task state for 4 min. During the resting-state recordings, the subjects were instructed to relax, stay awake, and keep their eyes open and fixed on the crosshairs on the screen. The details of the task state in this experiment are described in a previous paper [35]. Grayscale pictures of 24 unfamiliar faces (12 males and 12 female) were employed as stimuli. They were divided into three groups: negative emotion (angry, disgusted, or sad), positive emotion (happy), and neutral emotion. Ten healthy controls rated each face by emotion type and intensity. The probabilities that the emotional faces were correctly discriminated was 98% and 99% for the negative and positive emotion faces, respectively. The task had a block design with face processing and control conditions. Under the face processing condition, the subjects were instructed to discriminate the emotional face between two faces displayed on the screen, one emotional and the other with a neutral emotion (Figure 1). Under the control condition, the subjects were shown two squares of different sizes and instructed to choose the larger one. A subject indicated his choice by pressing a button, using his right-hand fingers. Each of these discrimination tasks lasted 2.5 s, which were conducted eight times per block (20 s/block). In one 4 min task, there were 12 blocks: six emotional face condition blocks (described above) and 6 control condition blocks. The condition blocks were presented alternately throughout the task. We measured mRT in each of the emotional face condition blocks, control condition blocks, and emotional face + control condition blocks in the above-mentioned task with correct responses.

### 2.4. Definition of Regions of Interest (ROIs) and Calculation of PLE

First, we analyzed the fMRI data acquired during the face processing task to compare between the emotional face condition and the control condition using the fMRI Expert Analysis Tool (FEAT) Version 6.0, which is part of FMRIB's Software Library (FSL, http://www.fmrib.ox.ac.uk/fsl) for extracting the ROIs in the gray matter associated with face processing. For prestatistical processing, we performed motion correction using MCFLIRT [36], nonbrain region removal using BET [37], spatial smoothing using a Gaussian kernel corresponding to a FWHM of 8 mm, mean-based intensity normalization of all volumes using the same factor, and high-pass temporal filtering (Gaussian-weighted LSF straight line fitting, with sigma = 100 s). Moreover, the acquired fMRI images were nonlinearly registered to the MNI-152 template. Time-series statistical analysis was carried out using FILM [38] with local autocorrelation correction. Individual subjects' *Z* (Gaussian transformed) statistical images were thresholded using clusters determined at *Z* > 4 and a whole-brain (corrected) cluster significance threshold of *p* = 0.05. Furthermore, only gray matter regions with voxels sizes (*n*) > 90 in significant clusters identified by the above-mentioned FSL analysis were used as the ROIs of this study. The percent BOLD signal changes between emotional face and control conditions were calculated in the ROIs in the gray matter regions associated with face processing and identified by the above FSL analysis, namely, the medial prefrontal cortex (mPFC), posterior cingulate cortex (PCC), amygdala (AMYG), and fusiform face area (FFA), using the AFNI program (3dDeconvolve) [39].

**Figure 2:**
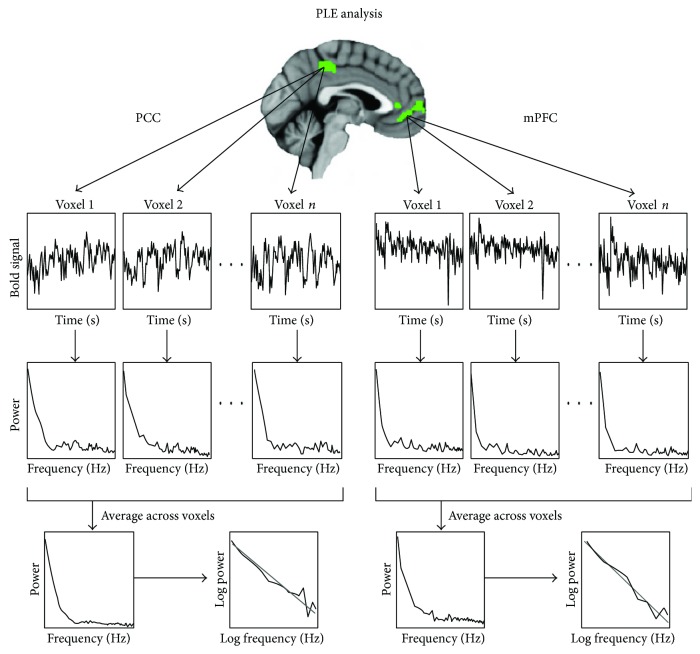
Methods of calculating PLE from BOLD signals with time course.

Then, we calculated PLE from the 4 min resting- and task-state fMRI data in each of the ROIs in mPFC, PCC, AMYG, and FFA (Figure 2). PLE is *β*, calculated from the formula power ∝ 1/frequency *β* [1–4, 6–8, 16]. Thus, PLE is the slope of the linear regression of the log power and log frequency of the power spectrum of BOLD signals. After the prestatistical processing, the time course per voxel was normalized to the zero mean and unit variance (*z* value) [40]. Because the variance is equal across all ROIs, all spectra have the same integrated area. Using methods previously optimized for fMRI [41], the normalized power spectrum of fMRI signals was computed for each voxel using the AFNI program (3dPeriodogram). The power spectrum of fMRI signals was further smoothed using a Hamming window of 7 (HM = 7) neighboring frequency bins. Additionally, we performed smoothing using other Hamming window sizes (HM = 3 and 5), to test the robustness of our results, as described in a previous paper [16].

First, using the fast Fourier transform, power and frequency were computed from BOLD signals with the time course of each voxel within the ROI, that is, mPFC, PCC, AMYG, and FFA. Secondly, the obtained power spectra were averaged across voxels within a certain ROI. Then, PLE was defined as the slope of the linear regression of log power on log frequency. In the next step, the power spectra averaged across voxels within each of the ROIs were extracted for each subject. A power spectrum was fitted with a power-law function P∝1/f*β* by least-square estimation (in a log frequency by log power plot) at frequencies of <0.1 Hz [2]. Finally, the power-law exponent *β* of each subject's ROI was defined as the slope of the linear regression of log power on log frequency corresponding to the straight-line regime.

### 2.5. Statistical Analyses

To assess task difficulty, the paired Student *t*-test was applied to both percent correct responses and reaction time under the control and face processing conditions. To assess the relationship between mRT in the face processing task and PLE in each of the ROIs, namely, mPFC, PCC, AMYG, and FFA, Pearson's correlation test was performed. The paired Student *t*-test was used for the comparison of PLE between the resting and task states. A statistical threshold of *p* < 0.05 was used.

## 3. Results

### 3.1. Percent Correct and Reaction Time Duration Difference between Control and Face Processing Conditions

The paired Student *t*-test showed significant differences in both percent correct responses and reaction time duration between control and face processing conditions (*p* < 0.01).

### 3.2. Task-Evoked Activity Differences between Face Processing and Control Conditions

**Figure 3:**
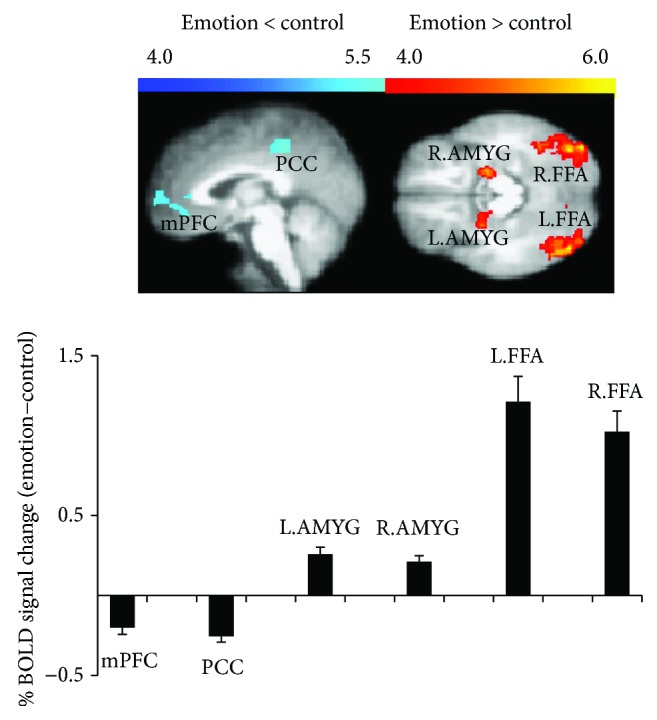
BOLD signal changes under face processing and control conditions. Changes are detected in mPFC, PCC, AMYG, and FFA.

The face processing condition showed a significantly decreased BOLD signal intensity in mPFC [(*x*, *y*, *z*) = (4, 44, −2); voxel size = 209; maximum *Z*-score = 4.98] and PCC [(*x*, *y*, *z*) = (2, −32, 44); voxel size = 170; maximum Z-score = 5.52]. In contrast, a significantly increased task-evoked BOLD signal intensity was observed in bilateral AMYGs [left, (*x*, *y*, *z*) = (−20, −6, −16); voxel size = 106; maximum Z-score = 5.36, and right, (*x*, *y*, *z*) = (26, −2, −14); voxel size = 99; maximum Z-score = 5.16] and bilateral FFAs [left, (*x*, *y*, *z*) = (−38, −50, −18); voxel size = 1421; maximum Z-score = 5.96, and right, (*x*, *y*, *z*) = (44, −54, −20); voxel size = 1399; maximum Z-score = 6.02], as compared with the control condition (Figure 3).

### 3.3. Comparison of PLE between Resting and Task States

**Figure 4:**
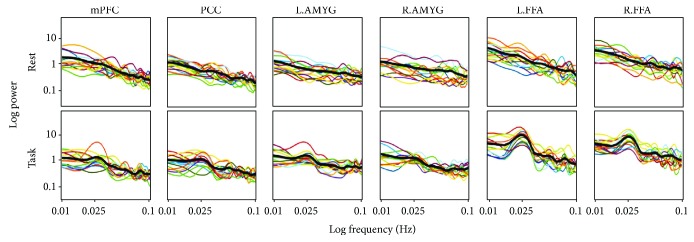
Slope of linear regression of log power on log frequency for HM = 7. Each slope of linear regression of log power on log frequency corresponding to each subject enrolled in this study is shown for mPFC, PCC, AMYG, and FFA for HM = 7. The black lines indicate average values for each brain region.

**Figure 5:**
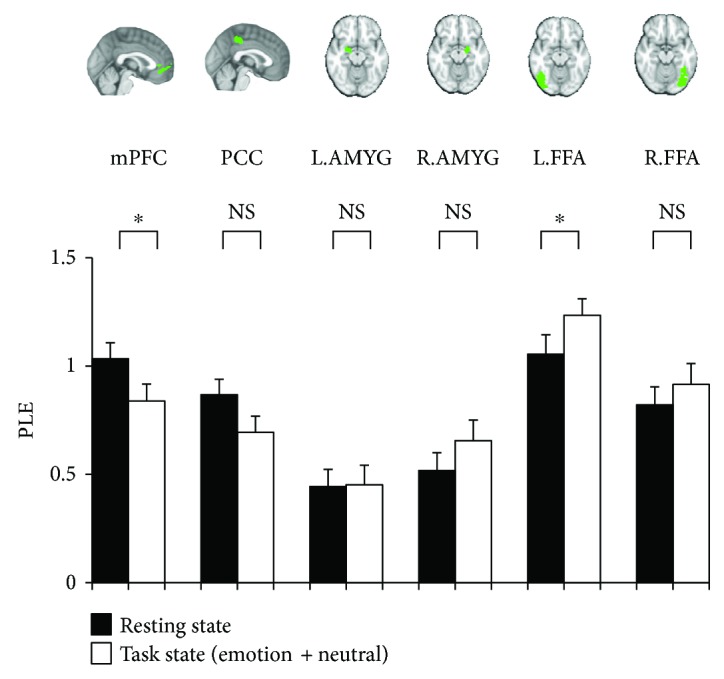
PLEs in resting state and task state for HM = 7. PLEs determined from fMRI images of mPFC, PCC, AMYG, and FFA using HM = 7. The paired *t*-test was used. ^∗^*p* < 0.05.

The log transformation of the power spectrum for HM = 7 is shown in Figure 4 as the main data. PLE in the task state was significantly lower in mPFC than in the resting state (*p* < 0.05). In contrast, PLE in the left FFA was significantly higher in the task state than in the resting state. Finally, the other regions such as PCC, left and right AMYGs, and right FFA did not show any significant difference in PLE between the resting state and the task state (Figure 5). Moreover, the additional analyses using HM = 3 and 5 showed that in mPFC, PLE in the task state was significantly lower than that in the resting state (*p* < 0.01 and *p* < 0.05, resp.), and in left FAA, PLE in the task state was significantly higher than that in the resting state (*p* < 0.05, resp.). Furthermore, the other regions such as PCC, left and right AMYGs, and right FFA did not show any significant difference in PLE between the resting state and the task state.

### 3.4. Relationship of mRT and PLE with Percent BOLD Signal Changes in Resting State, Task State, and Resting-Task Difference

**Figure 6:**
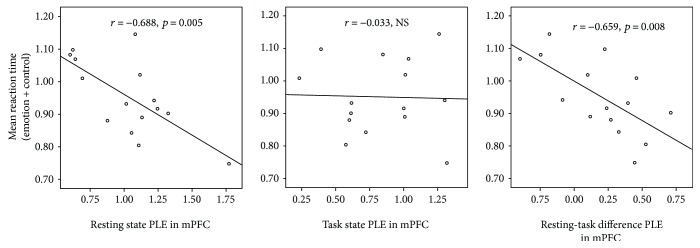
mRT and PLE in resting state, task state, and resting-task difference in mPFC for HM = 7. Pearson's correlation analysis (1000-sample bootstrapping) showed that in mPFC, mRT under the emotional face + control conditions was significantly negatively associated with PLE in the resting state (*r* = −0.688; 95% CI: –0.239 to −0.902; *p* = 0.005).

Pearson's correlation analysis (1000-sample bootstrapping) showed that in mPFC, mRT under emotional face + control conditions was significantly negatively associated with PLE in the resting state (*r* = −0.688; 95% CI: −0.239 to −0.902; *p* = 0.005). Moreover, mRT under the emotional face + control conditions also correlated with the resting-task difference PLE in mPFC (*r* = −0.659; 95% CI: −0.352 to −0.869; *p* = 0.008). In contrast, no significant correlation was found between task-state PLE in mPFC and mRT (Figure 6). Also, additional analyses using HM = 3 and 5 showed that in mPFC, the mRT in the emotional face + control conditions was significantly negatively association with the PLE of the resting state (HM = 3, *r* = −0.676; 95% CI: −0.211 to −0.900; *p* = 0.006, and HM = 5, *r* = −0.682; 95% CI: −0.240 to −0.900; *p* = 0.00, resp.). Moreover, mRT under the emotional face + control conditions was significantly negatively association with the resting-task difference in mPFC (HM = 3, *r* = −0.660; 95% CI: −0.376 to −0.867; *p* = 0.007, and HM = 5, *r* = −0.657; 95% CI: −0.372 to −0.867; *p* = 0.008, resp.). The analyses using both HM = 3 and 5 showed no significant correlation between task-state PLE in mPFC and mRT. Unlike in mPFC, we observed no correlation of mRT with PLE in the resting state, task state, and resting-task difference in the other regions, namely, PCC and bilateral AMYGs and FFAs in all analyses using HM = 3, 5, and 7. On the other hand, mRT under each of emotional face condition or control condition showed no significant association with resting-state PLE, task-state PLE, and resting-task difference PLE for all ROIs.

Finally, we found no significant correlation of percent BOLD signal changes with mRT in all the regions. In addition, Pearson's correlation analysis showed no significant correlations of age, years of education, or predicted IQ with any of the measures above, for example, percent BOLD signal changes, resting-state PLE, task-state PLE, and resting-task difference PLE for all ROIs.

## 4. Discussion

Here, we investigated the relationship between scale-free dynamics, in terms of PLE, in the resting and/or task state and the behavioral performance in the face processing task. Our findings in mPFC show the following: (i) a significant decrease in PLE in the face processing task compared with that in the resting state, (ii) a significant correlation between resting-state PLE and mRT under the emotional face + control conditions, and (iii) a significant correlation between resting-task difference PLE and mRT under the emotional face + control conditions. Taken together, these findings underline the central relevance of scale-free dynamics in the resting state and task-evoked activity to task performance parameters (e.g., mRT).

In the analysis of the BOLD signal changes under the face discrimination condition relative to the control condition in this study, AMYG and FFA were activated whereas mPFC and PCC were deactivated. These findings are in agreement with recent brain imaging findings on facial emotion processing [42, 43]. The deactivation we observed in midline regions such as mPFC and PCC has also been observed in other studies on emotion and cognitive processing [44]. We further observed a task-evoked decrease in PLE specifically in mPFC, which is in agreement with the previous finding of PLE modulation by sensory and motor tasks in the high-frequency ranges, as measured mostly by EEG/MEG [2, 7, 18–20]. Those previous findings together with our results suggest that the PLE decrease from the spontaneous state to the task state is observed in not only brain areas corresponding to sensory and motor functions but also other areas such as those involved in face processing. Moreover, our findings show differential PLE modulation in different regions, that is, mPFC showed a task-evoked decrease in PLE whereas PCC showed no such decrease. This differential PLE modulation observed in this study suggests specific roles of mPFC in mediating PLE modulation and the task-evoked modulation of PLE during the face processing task, which is in accordance with the previous findings that PLE in mPFC is involved in mediating emotions [45] and self-consciousness [16]. A high PLE suggests greater dependence of future dynamics on past dynamics within a range of low frequencies [2, 46]. In contrast, a decrease in PLE from the spontaneous state to the task state in mPFC may indicate less temporal dependence of future dynamics on past dynamics and a higher efficiency in current information processing required during task-evoked activity. Our assumption based on these results is that the higher PLE in the resting state and the lower PLE in the task state may result in higher efficiency in the processing of a task. This assumption is supported by the negative correlations of resting-state PLE and resting-task difference PLE with mRT.

Additionally, FFA showed an increase in PLE during task-evoked activity compared with the resting state activity, which indicates that FFA is likely required in face processing but not under the control condition [47]. The task-evoked PLE decrease is equivalent to the ratio of slow 5 (low frequencies, 0.01 to 0.027 Hz) to slow 4 (high frequencies, 0.027 to 0.073 Hz) shifting to more slow 4. Therefore, the task-evoked PLE increase in FFA may be related to this shift towards slow 5 relative to slow 4. Whether such a relative power shift in slow 5 may be related to the block paradigm with blocks of 20 s and emotional blocks occurring every 20 s remains unclear however. Our task-evoked PLE in the FFA showed an increase in power specifically every 40 s, which corresponds to 0.025 Hz, which is due to our block design; this suggests that the task-evoked PLE in FFA somewhat encoded the temporal structure of the paradigm. This remains to be a tentative thought and should be addressed in future studies.

In this study, we observed that resting-state PLE in mPFC inversely correlated with mRT under the emotional face + control conditions: the higher the PLE in the resting state, the shorter the mRT during the task. An analogous relationship was observed in the PLE difference between the resting and task states in this same region. On the other hand, PLE during the task-evoked activity itself, independent of the resting state, showed no relationship with task performance parameters, for example, mRT. This suggests that in mPFC, interindividual variability in mRT is related to interindividual variability in the scale-free dynamics in the spontaneous state (e.g., resting-state PLE) and the degree of task-evoked modulation from the scale-free dynamics in the spontaneous state to those in the task state.

Here, we mention the limitations of our study. We acquired MRI data for 4 min to calculate PLE both in the task and resting states, which is relatively short for determining PLE with sufficient S/N ratios. We set this duration because we had to acquire data for a moderate duration to weaken any effect of habituation during this face processing task. FFA exhibits task-evoked periodicity, as shown in Figure 4, owing to the block-task design in task-state fMRI. Although we discussed about the effect of this periodicity on our results, there still remain some issues. To manage this periodicity furthermore is very interesting but this is not within the scope of our study. We did not include any psychophysiological measures nor subjective measures, such as arousal, valence, or dominance, in the measurement of emotion-related effects in our study. The time series of these measures could have by themselves (such as heart rate) been analyzed for power-law distribution and then correlated with neuronal measures, carried out in previous EEG/MEG studies [7, 19, 20]. Moreover, one may point out that our task used to analyze PLE in this study included both emotional and control conditions; although our ROIs were based on face-processing-related effects (face versus control), the PLE measurement itself had to include both face processing and control conditions. Furthermore, in this study, significant differences were observed in percent correct response and reaction time between control and emotional face conditions, suggesting that the emotional face task is more difficult than the control task. Since a previous investigation revealed that the difficulty level of a task can contribute to PLE attenuation [48], the difference in task difficulty between control and emotional face conditions might affect on our results. Therefore, in future studies, paradigms with specific emotional conditions separate from control conditions should be designed, for example, nonemotion blocks, and there should be a balance in difficulty between task and control blocks.

In summary, we demonstrated that both resting-state PLE and resting-task difference PLE in mPFC were associated with task performance parameters, for example, mRT. This finding suggests a specific role of the scale-free dynamics of spontaneous brain activity in mediating task-evoked activity and behavioral performance in the face processing task. Our results complement previous studies showing the relevance of scale-free dynamics in perception, action, and cognition. This study further underlines the close relationship between spontaneous and task-evoked activities as shown by how scale-free dynamics mediate the transition of these processes and impact the associated behavioral performance.

## Figures and Tables

**Table 1:** Demographic characteristics of study subjects.

	Male
Number	15
Age (y)	24.1 ± 2.5
Education (y)	16.0 ± 0.7
Face processing task	
Reaction time under emotional face condition (s)	1.2 ± 1.6
Percent correct under emotional face condition (percent)	94.9 ± 4.6
Reaction time under control condition (s)	0.7 ± 1.4
Percent correct under control condition (percent)	99.7 ± 0.9
